# Prevalence of Coronary Artery Ectasia with Atherosclerosis and Associated Risk Factors in the West of Iran: A Cross-Sectional Study

**Published:** 2016-03-13

**Authors:** Farnaz Fariba, Mehdi Moradi, Arezzo Arabi, Behzad Ghaderi

**Affiliations:** ^a^ Department of Cardiology, School of Medicine, Hamadan University of Medical Sciences, Hamadan, Iran; ^b^ Clinical Research Development Unit of Farshchian Hospital, Hamadan University of Medical Sciences, Hamadan, Iran

**Keywords:** Ectasia, Coronary Artery, Atherosclerosis

## Abstract

**Background:** According to the angiographic findings, 3%-8% of atherosclerotic coronary artery patients suffer from coronary artery ectasia (CAE). We conducted this study to estimate the prevalence of CAE among patients who underwent angiography and compared this group with those patients without CAE and atherosclerosis in terms of common coronary heart disease (CHD) risk factors.

**Methods:** This cross sectional study was conducted in Hamadan Province, western Iran, from March 2014 to March 2015. Data were collected from angiography evaluation and clinical records in Ekbatan Hospital. The patients with atherosclerosis who had CAE were compared with patients who had neither CAE nor atherosclerosis. The categorical variables were compared using chi-squared test or Fisher’s exact test.

**Result:** Of 2767 patients who underwent coronary angiography, 166 (6.0%) had CAE with atherosclerosis, 2357 (85.2%) had atherosclerosis without CAE, and 244 (8.8%) had normal coronary artery. Compared to normal group, CAE patient were more hypertensive and smoker. Besides, in CAE group the proportion of dyslipidemia was higher than normal subject.

**Conclusions:** The prevalence of CAE in Hamadan Province was in the expected level. Distribution of common CHD risk factors were most prevalent in CAE patient in comparison with normal coronary artery group.

## Introduction

Cardiovascular diseases (CVD), particularly coronary heart disease (CHD) are the major leading cause of death in developing countries^1^. Mostly, manifestation of coronary artery involvement is stenosis but in some cases, abnormal coronary dilation or ectasia can occur^2^. The term of ectasia was first used by Bjork in 1966 to identify coronary vasodilatation^3^. Coronary artery ectasia (CAE) is a consequence of atherosclerosis in 50% of cases, whereas 20-30% of subjects have origin of fetus periods. According to the angiographic findings, 3%-8% of atherosclerotic coronary artery patients suffered from ectasia^4,5^.

CAE is defined as dilation of arteries, at least 1.5 times greater than the diameter of the adjacent normal segment, localized or widespread^5^. Markis et al. categorized CAE based on the extent of coronary involvement in four types: type I, diffuse ectasia of two or three vessels; type II, diffuse disease in one vessel and localized disease in another vessel; type III, diffuse ectasia of one vessel only; and type IV, localized or segmental ectasia^6^. Definitive mechanism of CAE is unknown, but a combination of genetic causes, common risk factors for coronary artery disease and abnormal metabolisms, involved in the development of CAE^7^. Yetkin et al. conducted a systematic review study to determine the common cause and mechanism of cellular, molecular and vascular involved in pathobiology of CAE and found that CAE was a systemic vascular wall abnormality rather than a simple variation of atherosclerosis^8^. Ectasia is more common in elementary and middle parts of right coronary artery (RCA) and then branch anterior descending (LAD) involvement^9^.

Despite increasing of knowledge on the risk factors of CAE, better understand of the associated factors affecting the atherosclerotic patient, particularly in developing countries is necessary. Numerous studies have been conducted in worldwide to investigate the prevalence of ectasia, but hitherto no study has been done in Hamadan Province. Hence, we conducted this study in order to, firstly, get more knowledge and determine of prevalence of the CAE amongst patients who underwent coronary angiography and comparison the distribution of common CHD risk factors in atherosclerotic patient suffer from CAE with normal coronary arteries subjects.

## Methods

This cross sectional study was conducted in Hamadan Province, western Iran, from March 2014 to March 2015. Overall, 2767 patients undergoing coronary angiography, for different reasons, in Ekbatan Hospital, were assessed. The most important indication for coronary angiography was positive noninvasive diagnostic tests, acute myocardial infarction (MI) and history of angina. CAE was defined as dilation of a segment of a coronary artery to a diameter of at least 1.5 time that of normal adjacent segments ^10^. According to the findings of angiography, the subjects were assigned in two groups: atherosclerotic patient with ectasia and subjects with normal coronary arteries. The existence of CAE in the major branches and also in the left main coronary artery (LM) was evaluated.

The local Human Subject Review Board of Hamadan University of Medical Sciences approved this study.

Cases identified by cardiologist were selected and demographic and clinical data were evaluated through patient records and examinations. Data collection tool was a researcher develop checklist including age, sex, hypertension (HTN), diabetes, cholesterol, smoking status and family history. Our definition of traditional risk factor for CHD disease included diabetes mellitus, hypertension, hypercholesterolemia and hypertriglycemia according to the American Heart Association definition. Thus patient who had a fasting plasma glucose concentration >125 mg/dL at two separate measurement, or used anti diabetic medication such as insulin or oral hypoglycemic agents considered as diabetic patient. Patients were considered as hypertensive if their mean systolic blood pressure was >140 mmHg or mean diastolic pressure was>90 mmHg or they were taking antihypertensive drug. Hypercholesterolemia was defined as cholesterol level>200 mg/dL and hyper triglyceride as triglyceride level >150 mg/dL or patients who were advised to use cholesterol / triglyceride- lowering drugs by their physician. Patients considered as smoker if they were using smoke in every rate. Comparison group were normal subjects undergoing angiography because CHD related conditions but no diagnosis of atherosclerosis and CAE.

For data analysis we used descriptive statistics including frequency tables, measures of central value and measures of dispersion to describe the study variables. Categorical variables were presented as number of cases (percentage) and were compared by the Χ^2^ test or Fisher’s exact test, when appropriate. SPSS version 16.0 (Chicago, IL, USA) used for all statistical analyses.

## Results

**Figure 1:**
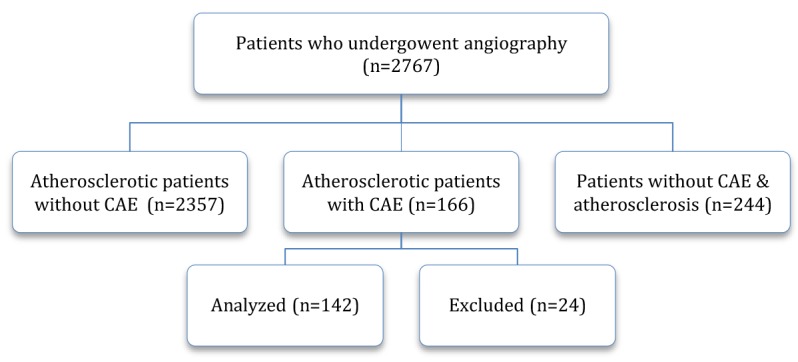
Algorithm of the study design

We excluded patients that availability of information was not possible (Figure 1). Overall, 2524 patients referred for coronary angiography, remained in the study. Of these, 166 (6.5%) atherosclerotic patients had CAE and 243 (9.6%) patients had normal coronary. The mean age of CAE patients was 59.4 ±1.19 yr (range 28 to 84 yr), and mean age of subjects with normal coronary artery was 58.50±9.8 yr (range 34 to 82 yr). From all patients with CAE, there were 57.7% hypertensive, 12.7% diabetic, 33.1% smoker and 59.9% had dyslipidemia (28.9% had high triglycerides and 31% had LDL). Moreover, 9.9% of cases had history of CHD disease in the family. 11.1% of CAE group and 10.6% of normal subject had positive family history of CAE but the difference was not statistically significant. The distribution of the clinical characteristics in patient with CAE and normal coronary is shown in Table 1.

**Table 1:** Characteristics of the patients with coronary artery ectasia (CAE) versus patients with normal coronary artery and atherosclerosis

**Variables**	**Patients with CAE** **and atherosclerosis**		**Patients with normal** **coronary artery**		***P*** ** value**
	**Number**	**Percent**	**Number**	**Percent**	
Gender					0.526
Male	87	61.3	150	61.5	
Female	55	38.7	94	38.5	
Smoker					0.001
Yes	48	33.8	45	18.4	
No	94	66.2	199	81.6	
Positive family history					0.511
Yes	15	10.6	27	11.1	
No	127	89.4	217	88.9	
Diabetic					0.089
Yes	19	13.4	47	19.3	
No	123	86.6	197	80.7	
Hypertensive					0.001
Yes	82	57.7	45	18.4	
No	60	42.3	199	81.6	
Hypertriglyceridemia					0.001
Yes	41	28.9	26	10.7	
No	101	71.1	218	89.3	
Hypercholesterolemia					0.001
Yes	44	31.0	17	07.0	
No	98	69.0	227	93.0	

## Discussion

About 6% of patients with atherosclerosis had CAE. The prevalence of CAE amongst total population undergone coronary angiography was 5.9%. Majority of the CAE patients were male and hypertensive. Limited studies investigated prevalence of CAE in Iran. The prevalence of CAE was 4% and 4.3% in 2009 and 2010 in Mashhad, respectively ^11,12^. Amirzadegan et al. found a prevalence of 1.5% CAE compared with those with a normal coronary artery in subjects who underwent coronary artery angiography^13^. The results of our study in comparison with some other papers, revealed the high prevalence of CAE in patients under study because this proportion have been reported lower in international studies (Turkey 4.3%, Spanish 3.39%, Greek 2.7%, England 1.4% Singapore 1.2%,)^14-17^. This rate has been reported 3% to 8%^18^. Therefore, our findings are consistent with it^18^. There is a wide variation in the level of health of a society and lifestyle as well as differences in the prevalence of cardiovascular risk factors in the population under study. Reports of CAE in different publications depend on the methodology of study and availability of data and accuracy of information.

Male gender is an associated risk factor with occurrence of CAE^19,20^. A hospital based study showed male gender as an independent predictor of CAE ^15^. 91.2% of CAE subjects under investigation were male as well. Our results revealed that proportion of men with the CAE was more than women (61% vs. 39%), although this proportion in comparison with control group had not significant difference. In Tehran Heart Center study, CAE was remarkably predominant in male in compared with female patients (2.7% for men vs. 1.4% for women)^13^. Most of these studies illustrated that sex differences and high frequency CAE in men, are resulted from the nature of the disease and its association with atherosclerosis as well as the underlying cardiovascular disease, more prevalent in men.

Our results showed that no significant difference between mean age of patient who had CAE and normal group. This finding is inconsistent with previous reports, which determined CAE patient are older than other group. ^4,21^ Amirzadegan et al.^13^ performed a retrospective cohort study in Iran and indicated that the patients with CAE were significantly older than those with normal coronary arteries. Generally, age has not been identified as an important risk factor^22,23^. This issue that indicates a well-designed of analytical studies with more robust methodology is needed to measure the independent effect of age on occurrence of ectasy.

According to the findings, 13.5% of CAE patients had diabetes. Although our evidences did not show a significant difference in the proportion of diabetic patients in both groups, but there was much debate about diabetes and its association with ectasia. In the previous texts, diabetes was introduced as a risk factor for the occurrence of ectasia, but in recent years after new bimolecular researches suggests that diabetes may have a protective effect against CAE^24,25^. The prevalence of diabetes in studied patients with CAE was 8% to 33% and diabetes might play a protective role for the development of CAE (OR=0.65, 0.54, 0.77, *P*<0.001)^24^.

We found that approximately 60% of cases had dyslipidemia. Our results reveal a strong significant association between dyslipidemia including hypertriglyceridemia and hypercholesterolemia with CAE. Several previous studies have also reported dyslipidemia in CAE subjects as 63% in Singapore^17^, 59% in Saudi Arabia^26^ and 41.7% in Iran^13^. Clearly, there is consistency between the findings of these various studies, and our results also is in agreement with other studies showed dyslipidemia was very prevalent in CAE patient in vs. normal coronary group. Sudhir et al. conducted a study in USA on 197 asymptomatic subjects with hypercholesterolemia and found that ectasia was significantly associated with a lower HDL cholesterol level and a higher LDL/HDL ratio ^27.^

Hypertension (HTN), one of the most serious threat and risk factors, has been consistently correlated with increased probability of developing CAD in various populations. HTN is the most common risk factor in Iran, and 25% of Iranian people (25-64 yr old) suffer from HTN^29^. Based on our study, 57.7% of CAE subjects suffered from the degrees of hypertension and this proportion was significantly higher than normal coronary group. This proportion in a study on 10057 angiographic procedures among Iranian population was 52.6% ^13^. CAE was significantly correlated to hypertension so that the adjusted OR estimate of CAE in hypertensive subjects against no hypertension was 2.43(95% CI (1.46–4.13)^21^. However, most of studies have not shown a significant association between HTN and CAE and this causal effect remind up to the present time controversial.

We investigated the proportion of smoking status was significantly higher in CAE patient than in control group (33.8% vs 7.0%). This finding indicated that smoking as traditional cardiovascular risk factor in our study subjects is less than some other studies (Pakistan 37.0%, China 53.4%, Iran 53.7%)^13,21,30^. Smoking is a common risk factor in CAE cases due to the fact that CAE is common in men and likely to be smoker more than women. Accordingly, it seems that there is essentiality to run a prospective study and systematic review & meta-analysis study to determine the predisposing and precipitating factors such as diabetes, hypertension, dyslipidemia and etc. in order to estimate the effect of potential risk factors that may affect progression CAE in CAD subjects.

We did not have distribution of coronary ectasia in different vessels of patients. Despite its limitations, this paper is the first study to determine the prevalence of ectasia in the Hamadan Province that can provide basic information for analytical studies that must be performed in the future.

## Conclusions

The finding of this study identified the prevalence of CAE in Hamadan Province was in the expected level. We conclude that, distribution of common CHD risk factors was most prevalent in CAE patient. Conducting similar studies in other province of country would produce more knowledge of epidemiological and clinical factors affecting on CAE in Iran.

## Acknowledgments

Authors take sincere thanks to clinical research Centre of Ekbatan Hospital for their help and financial support.

## Conflict of interest statement

Another’s declare no conflict of interest

## References

[1] Gu D, Reynolds K, Wu X, Chen J, Duan X, Muntner P (2002). Prevalence, awareness, treatment, and control of hypertension in china. Hypertension.

[2] Li JJ, Nie SP, Qian XW, Zeng HS, Zhang CY (2009). Chronic inflammatory status in patients with coronary artery ectasia. Cytokine.

[3] Björk L (1966). Ectasia of the coronary arteries. Radiology.

[4] Satran A, Bart BA, Henry CR, Murad MB, Talukdar S, SatranD SatranD (2005). Increased prevalence of coronary artery aneurysms among cocaine users. Circulation.

[5] Giannoglou GD, Antoniadis AP, Chatzizisis YS, Damvopoulou E, Parcharidis GE, Louridas GE (2006). Prevalence of ectasia in human coronary arteries in patients in northern Greece referred for coronary angiography. Am J Cardiol.

[6] Markis JE, Joffe CD, Cohn PF, Feen DJ, Herman MV, Gorlin R (1976). Clinical significance of coronary arterial ectasia. Am J Cardiol.

[7] Lin TA, Chen CW, Lin TK, Lin CL (2008). Tzu Coronary Artery Ectasia J.

[8] Yetkin E, Waltenberger J (2007). Novel insights into an old controversy: is coronary artery ectasia a variant of coronary atherosclerosis?. Clin Res Cardiol.

[9] Nyamu P, Ajit MS, Joseph PK, Venkitachalam L, Sugirtham NA (2003). The prevalence and clinical profile of angiographic coronary ectasia. Asian Cardiovasc Thorac Ann.

[10] Hartnell GG, Parnell BM, Pridie RB (1985). Coronary artery ectasia Its prevalence and clinical significance in 4993 patients. Br Heart J.

[11] Ahmadi M. Prevalance of ectasia in angiographic patientst, referred to Imam Reza hospital. Mashad: Mashad University of Medical Sciences; 2009.

[12] Ghasemi Torshizi G. Evaluation of cardiovascular risk factors in one hundred patients with coronary artery ectasia. Mashad: Mashad University of Medical Sciences; 2010.

[13] Amirzadegan AR, Davoodi G, Soleimani A, Lotfi Tokaldany M, Hakki Kazazi E, Shabpiray H (2014). Association between traditional risk factors and coronary artery ectasia: a study on 10057 angiographic procedures among iranian population. J Tehran Heart Cen.

[14] Yilmaz H, Sayar N, Yilmaz M, Tangürek B, Cakmak N, Gürkan U (2008). Coronary artery ectasia: clinical and angiographical evaluation. Turk Kardiyol Dern Ars.

[15] Pinar Bermúdez E, López Palop R, Lozano Martínez-Luengas I, Cortés Sánchez R, Carrillo Sáez P, Rodríguez Carreras R (2003). Coronary ectasia: prevalence, and clinical and angiographic characteristics. Rev Esp Cardiol.

[16] Giannoglou GD, Antoniadis AP, Chatzizisis YS, Damvopoulou E, Parcharidis GE, Louridas GE (2006). Prevalence of ectasia in human coronary arteries in patients in northern Greece referred for coronary angiography. Am J Cardiol.

[17] Lam CS, Ho KT (2004). Coronary artery ectasia: a ten-year experience in a tertiary hospital in Singapore. Ann Acad Med Singapore.

[18] Bonow RO, Mann DL, Zipes DP, Libby P. Braunwald's Heart disease: A textbook of cardiovascular medicine. 10th ed. Philadelphia: Saunders; 2015.

[19] Almansori MA, Elsayed HA (2015). Coronary artery ectasia - A sample from Saudi Arabia. J Saudi Heart Assoc.

[20] Keçebaş M, Beşli F, Alişir MF, Çalışkan S, Yıldırım A, Ayd A (2015). The relationship between atherosclerotic risk factors and coronary artery disease in coronary artery ectasia. Duzce Medical Journal.

[21] Yang JJ, Yang X, Chen ZY, Wang Q, He B, Du LS (2013). Prevalence of coronary artery ectasia in older adults and the relationship with epicardial fat volume by cardiac computed tomography angiography. J Geriatr Cardiol.

[22] Sudhir K, Ports TA, Amidon TM, Goldberger JJ, Bhushan V, Kane JP (1995). Increased prevalence of coronary ectasia in heterozygous familial hypercholesterolemia. Circulation.

[23] Demopoulos VP, Olympios CD, Fakiolas CN, Pissimissis EG, Economides NM, Adamopoulou E, el at (1997). The natural history of aneurysmal coronary artery disease. Heart.

[24] Huang QJ, Liu J, Chen MH, Li JJ (2014). Relation of diabetes to coronary artery ectasia: A meta-analysis study. Anadolu Kardiyol Derg.

[25] Androulakis AE, Andrikopoulos GK, Kartalis AN, Stougiannos PN, Katsaros AA, Syrogiannidis DNN (2004). Relation of coronary artery ectasia to diabetes mellitus. Am J Cardiol.

[26] Almansori MA, Elsayed HA (2015). Coronary artery ectasia - A sample from Saudi Arabia. J Saudi Heart Assoc.

[27] Sudhir K, Ports TA, Amidon TM, Goldberger JJ, Bhushan V, Kane JP (1995). Increased prevalence of coronary ectasia in heterozygous familial hypercholesterolemia. Circulation.

[28] Lewington S, Clarke R, Qizilbash N, Peto R, Collins R (2002). Age-specific relevance of usual blood pressure to vascular mortality: a meta-analysis of individual data for one million adults in 61 prospective studies. Lancet.

[29] Esteghamati A, Abbasi M, Alikhani S (2008). Prevalence, awareness, treatment, and risk factors associated with hypertension in the Iranian population: the national survey of risk factors for noncommunicable diseases of Iran. Am J Hypertens.

[30] Sultana R, Sultana N, Ishaq M, Samad A (2011). The prevalence and clinical profile of angiographic coronary ectasia. J Pak Med Assoc.

